# Ileo-ileal knot causing small bowel obstruction and spontaneous fetal loss in a 25-week pregnancy: A case report

**DOI:** 10.1016/j.ijscr.2025.111753

**Published:** 2025-07-29

**Authors:** Brian Kasagga, Dan Ssekiwunga, Godfrey Kikuba, Odok Ambrosoli, Joel Mulema, Paul Okeny

**Affiliations:** a Department of Surgery, Makerere University School of Medicine, Uganda; b Society of Uganda Gastrointestinal and Endoscopic Surgeons (SUGES), Uganda; c Department of Radiology, Makerere University School of Medicine, Uganda

**Keywords:** Ileo-ileal knot, Intestinal obstruction, Pregnancy, Fetal loss, Case report

## Abstract

**Introduction and importance:**

Small bowel obstruction (SBO) during pregnancy is a rare but potentially life-threatening condition, with high maternal and fetal morbidity. Among the rarest etiologies is ileo-ileal knotting, a rarely encountered surgical emergency with only seven reported cases in pregnancy, including one ileo-ileal knot, as per Shimizu et al. (2014). Its timely diagnosis is often hindered by overlapping obstetric and gastrointestinal symptoms.

**Case presentation:**

We present the case of a 30-year-old woman at 25 weeks gestation who experienced severe abdominal pain, distension, and constipation for five days. Initially managed conservatively at a peripheral facility, she suffered a spontaneous fetal loss and was referred to a tertiary center. Exploratory laparotomy revealed an ileo-ileal knot with necrotic bowel, necessitating resection and primary anastomosis. Postoperative recovery was complicated by wound infection but resulted in full recovery with no long-term sequelae.

**Clinical discussion:**

Ileo-ileal knotting results in rapid bowel ischemia and necrosis due to vascular compromise. Risk factors may include pregnancy-related bowel displacement and predisposing anatomical anomalies such as adhesions or Meckel's diverticulum. Diagnosis is often delayed due to atypical presentation and limited access to imaging in low-resource settings. Early surgical intervention remains the cornerstone of management, as delayed treatment significantly increases morbidity and mortality.

**Conclusion:**

This case highlights the need for a high index of suspicion for rare causes of intestinal obstruction in pregnancy. Prompt surgical management can improve maternal and fetal outcomes. In low-resource settings, early clinical decision-making remains essential to preventing complications when advanced diagnostics are unavailable.

## Highlights

• This rare case of ileo-ileal knotting in pregnancy, probably the second documented case in medical literature.
• It highlights the challenges of managing bowel obstruction in pregnant patients, balancing urgency with fetal safety.
• Despite diagnostic delays and fetal loss, the patient recovered well after surgical resection and anastomosis.

## 1 Introduction

Small bowel obstruction (SBO) occurs in 1 in 1500 to 1 in 16,000 pregnancies and is among the top three surgical emergencies in pregnancy; caused by adhesions, hernias, and intestinal knots [1]. Diagnosing and managing acute abdominal pain in pregnancy is challenging due to the overlap of symptoms between obstetric and non-obstetric conditions. Despite its rarity, intestinal obstruction in pregnancy is associated with significant maternal and fetal mortality, making timely diagnosis and treatment crucial [2].

Among the causes of intestinal obstruction, Ileo-ileal knot is an extremely rare condition, and even more so during pregnancy. This occurs when the ileum rotates around the axis of its mesentery, resulting in vascular occlusion. A review by Shimizu et al. in 2014 highlighted only seven published cases of knotting in pregnancy, with just one involving an ileal knot; the rest were ileosigmoid knots [3].

We present a case of a 36-year-old woman who was referred to our tertiary hospital following a spontaneous fetal loss. She had experienced symptoms of intestinal obstruction for nearly a week. Upon operation, we found an ileal knot. Despite the unfortunate loss of pregnancy, and receiving definitive treatment rather later than would have been required, the patient had a good outcome and was discharged in good condition.

## 2 Methods

This case report has been reported in line with the SCARE criteria (Surgical CAse REport) 2025 guidelines, ensuring comprehensive and transparent reporting of the clinical details [4].

## 3 Case presentation

A 30-year-old female was referred from a higher-level center to our tertiary hospital for further management after presenting with a spontaneous pregnancy loss at 25 weeks. She had experienced severe abdominal pain for 6 days, abdominal distension for 5 days, and constipation for 5 days. The pain was colicky, generalized, of sudden onset, and persistent throughout the day. She reported no vomiting but was unable to pass flatus for 3 days. Initially, she sought care at a peripheral medical center, where she was treated for partial intestinal obstruction using a nasogastric tube and enemas, leading to the passage of soft stool and some symptomatic relief. An ultrasound scan which was done during this time showed a single live fetus at 25 weeks, with enlarged bowel in the right and left hypochondriac regions. No further investigations were conducted, and she was discharged after 3 days after presenting to this unit.

However, 18 h post-discharge, she experienced a spontaneous fetal loss at 25 weeks' gestation, prompting her to seek further care at a higher-level center. After initial stabilization for 3 h, she was then referred to our tertiary hospital. Notably, her obstetric history included two previous cesarean sections.

On examination, the patient was afebrile, temperature of 36.8 °C and had mildly dehydrated. Vital signs were as follows: blood pressure of 127/86 mmHg, pulse rate of 116 bpm (tachycardia), and SpO2 at 95 %. Abdominal examination revealed moderate distension, symmetrical movement with respiration, without visible collaterals, bulges, or peristalsis. Palpation showed moderate tenderness, and the symphysial fundal height was 22 cm. The uterus was well contracted and not boggy. Bowel sounds were hyperactive, occurring 8 times in 30 s. Vaginal examination revealed a normal vulva and vagina with a minimal amount of fresh blood. Rectal examination indicated a normal anal sphincter, no masses, and an empty rectal vault.

**Fig. 1:**
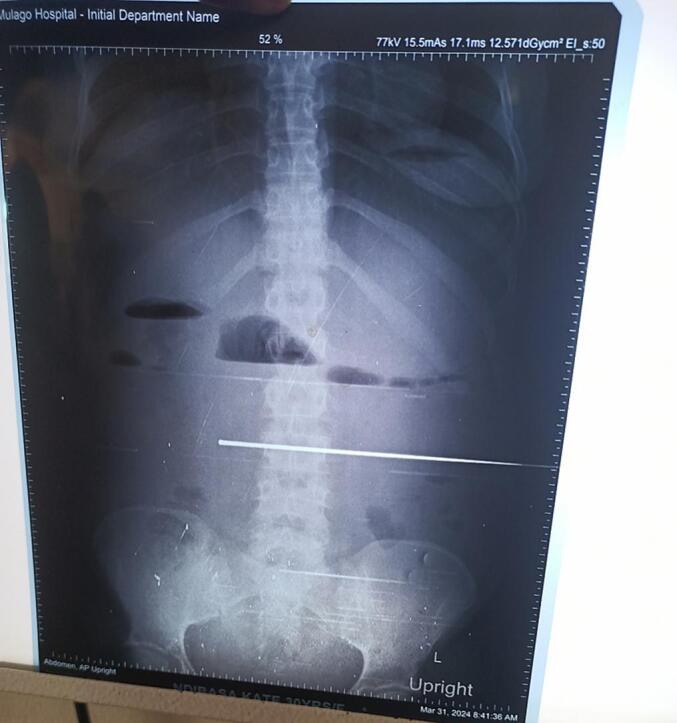
Erect Abdominal X-ray showing five air fluid levels.

Appropriate resuscitation was initiated with intravenous fluids (ringers' lactate), antibiotics (IV ceftriaxone), supplementary oxygen and analgesia, and a nasogastric tube was inserted for decompression. The patient was kept nil per os (NPO). Comprehensive blood workup, including a complete blood count (CBC), liver function tests (LFTs), renal function tests (RFTs), and blood grouping with cross-matching, was performed. An erect abdominal X-ray was ordered to confirm the diagnosis of Intestinal obstruction. (Fig. 1). Additionally, a urinary catheter was placed for monitoring urine output. The CBC showed a hemoglobin of 8.3 g/dl, White blood cell of 13,000 predominantly neutrophils (85 %), with normal RFTS, electrolytes and LFTs.

Given the patient's history, X-ray findings, and recent pregnancy loss, the findings were discussed with the surgical team and the on-call consultant. A decision was made to carry out an urgent laparotomy to ascertain and address the cause of the intestinal obstruction. Informed consent was obtained from the patient's husband because the patient was confused and agitated. The patient was then wheeled into the emergency department operating room. The procedure was performed by two surgical residents under general anesthesia in the night, approximately 12 h after presentation to the surgical emergency unit.

### 3.1 Intraoperative findings and surgical steps

**Fig. 2:**
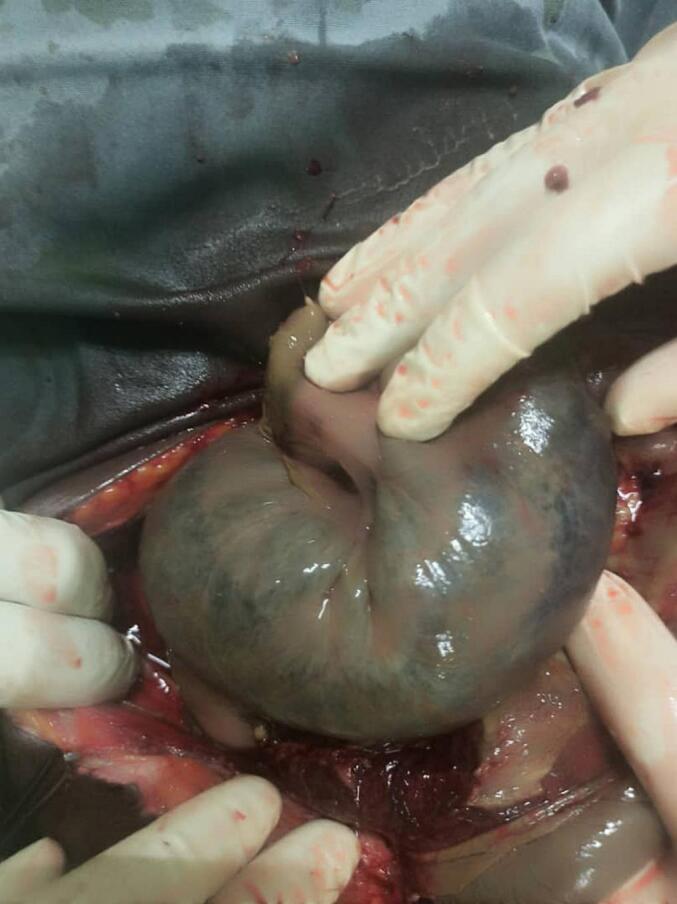
Necrotic ileum.

**Fig. 3:**
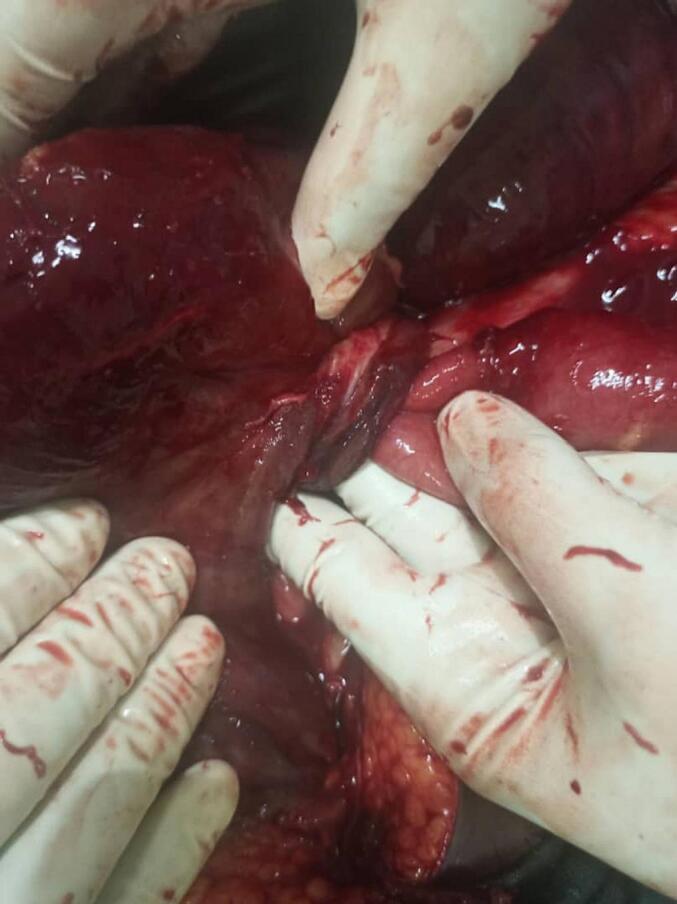
Showing the base of the knot, after retraction of the necrotic ileum.

**Fig. 4:**
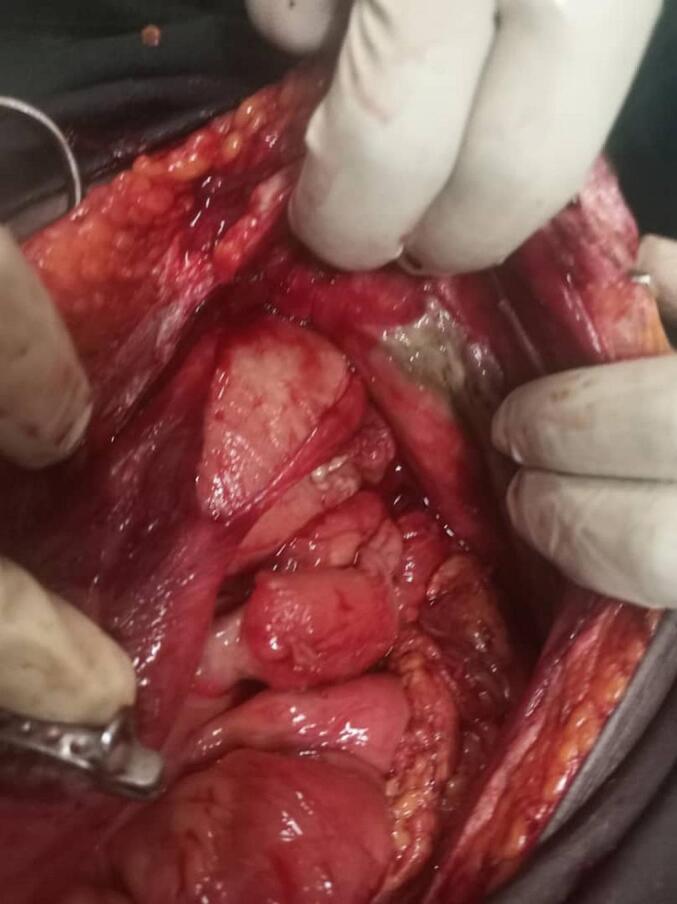
Showing an outpouching of bowel and adhesive band on the antimesenteric border that were tethering onto the abdominal wall. This outpouching was suspected intraoperatively to be a Meckel's diverticulum, though not histologically confirmed.

A laparotomy was performed using a midline incision under general anesthesia. We found approximately 1 l of brown, odorous ascitic fluid. We also found gangrenous small bowel which was twisted to form an anticlockwise knot (Fig. 2, Fig. 3). The knot was approximately, about 200 cm from the ileocecal junction. There was dilated bowel proximal to the knot. Lastly, the knot was tethered by an adhesive band and a 6 cm outpouching on the antimesenteric border of the ileum, both of which were attached to the abdominal wall (Fig. 4). We suspected the outpouching to be a Meckel's diverticulum and considered it, along with the adhesive band, as a potential axis of rotation. However, histopathological analysis was not performed, and thus this diagnosis remains presumptive. The uterus was well contracted. The adhesive band and outpouching were sharply dissected from the abdominal wall. Approximately 80 cm of necrotic small bowel, comprising of distal jejunum and proximal ileum, was resected en bloc along with the adhesive band and the outpouching. The ileocecal valve was identified and preserved. The bowel was milked to remove all stagnant contents. An end-to-end jejunoileal anastomosis was performed in two layers using 2/0 Vicryl. The abdominal cavity was then lavaged with warm saline, and a drain was placed in the right paracolic gutter.

### 3.2 Follow up and monitoring

Postoperatively, the patient received intravenous fluids, 2 units of packed red blood cells, analgesia, omeprazole, and intravenous Ceftriaxone and metronidazole, with regular monitoring. On the first postoperative day, she passed a soft stool, had reduced abdominal distension with audible bowel sounds, was afebrile, and had stable vital signs (PR 98 bpm, BP 129/66 mmHg). By the second day, she had no complaints, passed another bowel motion, and had a soft, non-tender abdomen; she was started on warm sips and had a WBC count of 12,000 and hemoglobin of 11.4 g/dl. On the fifth day, she began soft feeds. However, there was wound dehiscence and increased pus discharge from the wound, which prompted a pus swab for culture and sensitivity. At this point, antibiotics were changed to piperacillin/tazobactam and metronidazole, and the wound was dressed with iodine and vinegar. The abdominal drain was then removed as it was no longer functional. The discharge reduced and the wound formed good granulation tissue by 10th day. Secondary wound closure was done using Nylon 2/0 sutures on the 14th post operative day. She was then discharged on the same day and scheduled for review after a week.

A week after discharge, she returned and her wound had no discharge, and had properly healed.

## 4 Discussion

Ileo-ileal knot is a rare cause of strangulated intestinal obstruction, involving a closed loop phenomenon due to a knot in the mesentery, which can rapidly lead to bowel gangrene. This condition is associated with significant morbidity and has a high mortality rate of around 50 %. [3,[5], [6], [7]]

The etiology of ileo-ileal knotting is not well understood, but several risk factors have been suggested [6]. These factors might include a high-fiber bulky diet, excessive mobility of the ileum, and a redundant sigmoid colon [8]. Additional hypotheses for the development of ileo-ileal knotting include a small intestine that is longer relative to the mesentery, a hyperactive ileum, a capacious abdomen in thin individuals, postoperative status, single bulky meal intake with increased peristalsis, and the presence of adhesions. [6,7,9,10] Shimizu et al. postulated that pregnancy, especially in the third trimester, can displace the bowel, looping around each other and contribute to the formation of ileal knots [3]. In our patient, as seen in Fig. 3 above, the presence of a fibrous band and a possible Meckel's diverticulum, could have acted as an axis of rotation. Coupled with her pregnancy the displacement of bowel as described by Shimizu et al. above likely predisposed her to developing an ileal knot [3].

Once the knot forms, it initiates a vicious cycle of intestinal occlusion and ischemia. The knot causes vascular obstruction, and the elevated proximal pressures further interrupt the blood supply. Ultimately, these processes lead to gangrene [11]. Assessment of the patient will include taking appropriate history and appropriate investigations. Typical symptoms include abdominal pain, distension, vomiting, and obstipation [7]. However, diagnosing intestinal obstruction (IO) in pregnancy can be delayed for two reasons: firstly, the symptoms may not present typically, and secondly, changes like abdominal distension and vomiting can be mistaken for normal pregnancy symptoms. Additionally, concerns for the fetus often delay aggressive treatment, investigations or even surgery by preferring a conservative approach [8,9]. In our patient, this exactly occurred and conservative management with nasogastric tube decompression and enemas for over 3 days resulted in slight improvement but ultimately led to spontaneous fetal loss. The fetal loss may have been precipitated by maternal bowel ischemia and evolving sepsis due to the ileo-ileal knot, resulting in hemodynamic instability, systemic inflammatory cytokine release, anemia and subsequent reduction in uteroplacental perfusion [12]. Therefore, prompt work up and urgent surgical management of intestinal obstruction in pregnancy must always be done to prevent complications.

The diagnostic work-up in this case was limited by both clinical overlap and resource constraints. An abdominal ultrasound performed at the peripheral facility revealed a single live fetus at 25 weeks and dilated bowel loops in both the right and left hypochondriac regions. However, these findings were non-specific and did not lead to a definitive diagnosis. This is a common limitation of ultrasound in bowel obstruction due to interference by gas-filled loops [13]. An erect abdominal X-ray at our center showed multiple air-fluid levels, suggestive of small bowel obstruction.

While a contrast-enhanced CT scan could have provided definitive diagnostic information;- such as the characteristic “whirlpool sign” of twisted mesentery; − this modality could not be used [3]. CT scans involve ionizing radiation, which poses theoretical risks during pregnancy, including fetal growth restriction and neurodevelopmental effects at high doses [10]. Additionally, this diagnostic tool is expensive and often unavailable in many low-income settings [14]. Although fetal risk was no longer a concern following the loss, our patient was unable to afford a CT scan. We therefore relied on erect abdominal X-ray findings to confirm the diagnosis of Intestinal obstruction, and thus do an urgent exploratory laparotomy. These limitations highlight the need for high clinical suspicion and reliance on basic imaging modalities in resource-constrained environments.

Treatment should be initiated promptly, including aggressive IV fluid resuscitation, nasogastric tube insertion, and broad-spectrum antibiotics. After resuscitation, an emergency laparotomy should be performed. Intraoperative surgical decision making depends on the viability of the affected bowel segment. To prevent unnecessary bowel resection, some authors like Molla et al. recommend releasing the knot first to assess the amount of salvageable small bowel [8]. However, this can cause the movement of necrotic material and toxins into the circulation [15]. Other suggestions is if the bowel is nonviable, en bloc resection followed by anastomosis or exteriorization is preferred to avoid perforation and contamination, depending on the patient’s condition [7,8,15]. The patient's general condition will determine whether primary anastomosis or a stoma is required.

In our patient, a midline laparotomy was performed under general anesthesia approximately 12 h after arrival at the tertiary center. Intraoperative findings revealed a tight ileo-ileal knot with approximately 80 cm of gangrenous small bowel involving both distal jejunum and proximal ileum. En bloc resection of the necrotic segment was done, and a primary jejunoileal anastomosis was performed. Importantly, the ileocecal valve was preserved, which likely contributed to her favorable recovery and minimized risk of short bowel syndrome. Postoperative monitoring includes assessing hydration status, nutritional status, electrolyte balance, anemia, and signs of anastomotic leak [8]. This highlights the importance of not only intraoperative decision-making but also comprehensive postoperative care and nutritional follow-up.

Regarding follow-up, patients should be monitored for short bowel syndrome and malnutrition. Early management should include dietary modifications with complex carbohydrates and supplementation with essential vitamins (A, C, D, E, K) and micronutrients such as zinc and copper [16]. Preserving the ileocecal valve, when possible, helps reduce the risk of short bowel syndrome. This is achieved by slowing the transit time of intestinal contents, thereby allowing for more efficient absorption of nutrients and fluids. Additionally, the ileocecal valve plays a vital role in preventing the backflow of colonic bacteria into the small intestine, which helps to mitigate the risk of bacterial overgrowth and subsequent malabsorption. This preservation is essential for maintaining better nutritional status and quality of life in patients who undergo significant bowel resections [17,18]. In our case, preservation of the ileocecal valve may explain the patient's relatively smooth postoperative course and absence of major nutritional complications, despite the significant bowel resection.

This case compares with the previous study by Ucar et al. involving a 28-year-old woman who developed an ileal knot post-spontaneous vaginal delivery, presenting with hypovolemic and septic shock, abdominal compartment syndrome, and a markedly distended abdomen [19]. Unlike the patient in Ucar et al.'s case, who had no prior surgical history and experienced a rapid onset of symptoms immediately following labor, our patient presented with a history of two prior cesarean sections and had a more prolonged symptom onset over several days. Additionally, while Ucar et al.'s patient developed severe complications, including perforation, septic shock, and required multiple surgeries (resection of necrotic bowel, ileostomy with mucous fistula, and relaparotomy), ultimately succumbing to abdominal compartment syndrome, our patient, despite the delays in diagnosis, underwent successful surgical intervention involving the resection of necrotic ileum and primary anastomosis. This contrast underscores the variability in presentation and complications of ileal knots and highlights the critical importance of early diagnosis and prompt surgical treatment.

In conclusion, this case underscores the necessity of considering rare causes of intestinal obstruction, such as ileal knots, in pregnancy. The imperative for swift and decisive action to mitigate complications and improve patient outcomes is crucial, particularly given the high risk of maternal and fetal mortality. This highlights the importance of timely diagnosis and intervention in managing intestinal obstruction in pregnant patients to prevent adverse outcomes. Additionally, clinicians in low-resource settings should prioritize early surgical consultation and basic imaging (e.g., abdominal X-rays) when advanced modalities like CT are unavailable. Multidisciplinary collaboration between obstetricians, surgeons, and radiologists is critical to mitigate adverse outcomes. In similar environments, protocols for rapid referral and expedited laparotomy should be emphasized to reduce maternal and fetal risks.

## Ethical approval

Ethical approval is not required for case reports at our local hospital IRB.

## Sources of funding

There are no funding sources for this case report.

## Author contributions

Conceptualization: Brian Kasagga.

Methodology: Brian Kasagga, Odok Ambrosoli, Godfrey Kikuba, Dan Ssekiwunga.

Investigation: Dan Ssekiwunga, Godfrey Kikuba, Brian Kasagga.

Writing – Original Draft: Brian Kasagga, Joel Mulema, Godfrey Kikuba.

Writing – Review & Editing: Odok Ambrosoli, Brian Kasagga.

Supervision: Paul Okeny.

## Guarantor

Brian Kasagga.

## Research registration number

N/A

## Consent for publication

Written informed consent was obtained from the patient for publication of this case report and any accompanying images.

## Conflict of interest

The authors declare that they have no competing interests.

## Data Availability

For this case report, clinical information was obtained from patient files. On request, more details are available, but only in compliance with the hospitals' privacy rules.
